# Exogenous 6-benzylaminopurine enhances waterlogging and shading tolerance after anthesis by improving grain starch accumulation and grain filling

**DOI:** 10.3389/fpls.2022.1003920

**Published:** 2022-10-27

**Authors:** Wenjing Zhang, Beibei Wang, Anmin Zhang, Qirui Zhou, Yang Li, Lingyu Li, Shangyu Ma, Yonghui Fan, Zhenglai Huang

**Affiliations:** ^1^ Key Laboratory of Wheat Biology and Genetic Improvement on South Yellow and Huai River Valley, Ministry of Agriculture, Hefei, China; ^2^ College of Agronomy, Anhui Agricultural University, Hefei, China

**Keywords:** wheat (*Triticum aestivum* L.), waterlogging and shading, 6-benzylaminoadenine, photosynthetic performance, starch accumulation, grain filling, yield

## Abstract

Due to the frequent occurrence of extreme weather events, the area of wheat affected by continuous cloudy rainfall is increasing, with waterlogging becoming a major limiting factor of wheat yield. To alleviate the effect, spraying exogenous plant growth regulators is often used. In this study, two wheat cultivars, waterlogging-tolerant Yangmai 18 and waterlogging-sensitive Sumai 188, were selected for waterlogging and shading (WS) after anthesis for 7, 11, and 15 days respectively. Three concentrations of 6-benzylaminoadenine (6-BA) solution (15, 25, and 35 mg·L^−1^) were sprayed after WS treatment and water was sprayed as the control. Then, the effect of spraying 6-BA on photosynthetic characteristics, starch content, grain filling characteristics, and yield was explored under artificially stimulated continuous cloudy rainfall during anthesis. Compared with the control, the application of 6-BA caused a significant increase in grain plumpness throughout grain filling, as well as increases in the net photosynthetic rate (*P*_n_), stomatal conductance (*G*_s_), and transpiration rate (*T*_r_), and a significant decrease in the intercellular CO_2_ concentration (*C*_i_) of the flag leaves, all of which enhanced the photosynthetic capacity. The content of total starch, amylose, and amylopectin in the grains also increased significantly compared with the control. After WS for 15 days, the starch content increased by 3.81%–11.41% compared with the control. Spraying 6-BA also prolonged grain filling, increased the average grain filling rate, and significantly increased the 1000-grain weight and yield. The thousand-grain weight increased by 5.06%–43.28%, and wheat yield increased by 8.93%–64.27% after spraying 25 mg·L^−1^ of the 6-BA solution. These findings suggest that the application of 6-BA after WS stress could significantly improve the photosynthetic performance, which is propitious to the accumulation and transport of photosynthetic products after anthesis. Besides, spraying 6-BA can also increase the duration and rate of grain filling and starch accumulation content and improve grain weight, thereby alleviating the adverse effects of WS on wheat yield. Overall, spraying 25 mg·L^−1^ of the 6-BA solution had an optimal effect. These findings provide a theoretical basis for the exploration of cultivation techniques and measures aimed at alleviating damage caused by continuous rainfall during wheat anthesis.

## 1 Introduction

In the recent years, continuous or heavy rainfall caused by climatic factors, coupled with poor irrigation and drainage facilities as well as a low-lying terrain, has had widespread effects on global wheat production, with waterlogging becoming a major limiting factor of wheat yield (Boru et al., 2001; Singh and Setter, 2015). This is particularly apparent in the wheat-growing regions of the middle and lower reaches of the Yangtze River, China, where rice–wheat rotation has resulted in heavy soil with saturated water content (Shao et al., 2013). Moreover, much of the high rainfall in this region occurs in spring, a critical wheat growth period in terms of booting and anthesis. Studies have shown that the frequency of waterlogging in wheat-growing areas along the Yangtze River was as high as 60% during the 50-year period from 1961 to 2010, with detrimental effects on wheat yield (Wu et al., 2016; Wu H. et al., 2018). At the same time, the weak light effect caused by continuous precipitation has an additional effect during later growth stages, greatly reducing grain dry matter accumulation, which also affects yield (Mu et al., 2010). However, waterlogging stress and shading stress on the damage mechanism of wheat are different (Geigenberger, 2003; Gao et al., 2017). At present, there are many studies on the effects of single-factor stress such as waterlogging or shading, while there are few studies on the effect of compound stress on wheat yield. Under natural conditions, waterlogging is generally caused by continuous rainy weather, which leads to insufficient light. Therefore, the study of the combined stress of WS can better simulate the natural disaster situation of wheat. New cultivation techniques and measures aimed at effectively alleviating the damage caused by the combined stress of WS are therefore essential.

About 70% of wheat grain yield is associated with the accumulation of photosynthetic products after anthesis (Ziaei and Sepaskhah, 2003). Under waterlogging stress, the nitrogen content of wheat plants is significantly reduced, leaves become chlorotic, senescence is accelerated, and plants lose their ability to capture light and carboxylate (Ziaei and Sepaskhah, 2003; Wu W. et al., 2018). This has a direct effect on the rate of photosynthesis, dramatically slowing growth and development. Meanwhile, studies have also shown that while mild shading (88% light transmittance) delays the senescence of wheat leaves, increasing the *P*_n_, canopy apparent photosynthetic rate, 1000-grain weight and yield, and moderate (67% light transmittance) and heavy shading (35% light transmittance) has significant negative effects on all of the above traits (Xu et al., 2015). The later stages of wheat growth and development center on the formation of grains, the main component of which is starch, which accounts for about 65% of the dry weight. Starch in the wheat endosperm is composed of amylose and amylopectin, with differences in the composition having an important impact on flour quality (Golay et al., 1991; Holm and Björck, 1992). Waterlogging during heading and anthesis causes damage to endosperm cell structure, resulting in the formation of irregular starch granules (Zhou Q. et al., 2018a), in addition, post-anthesis waterlogging affects the content of starch components by altering the activity and expression of enzymes related to starch synthesis (Zhou Q. et al., 2018b). Similarly, shading treatment in the wheat filling stage also causes a decrease in the starch content of wheat grains, significantly reducing overall quality (Andrade et al., 2018). In addition, shading also causes a reduction in dry matter accumulation before anthesis and wheat yield (Demotes-Mainard and Jeuffroy, 2004; Mu et al., 2010).

The exogenous application of plant growth regulators is often used to alleviate the impact of water or shading stress on crops. For example, studies have shown that the application of abscisic acid (ABA) before waterlogging can improve the antioxidant and photosynthetic capacity of waterlogged crops (Kim et al., 2018). Application of methyl jasmonate increased grain yield and biomass and water use efficiency under water stress during the vegetative stage (Ghasem et al., 2022). It was also shown that exogenous application of salicylic acid (SA) inhibited the uptake of Na and stimulated the uptake of N, P, and K in wheat under water stress, decreasing stomatal conductivity and increasing the grain number per spike and 1000-grain weight (Hafez and Farig, 2019). 6-Benzylaminoadenine (6-BA) promotes cell division and the transport and accumulation of photosynthetic products, enhancing stress resistance in plants. Moreover, in comparisons of five hormones (auxin, cytokinin, abscisic acid, gibberellin, and brassino steroid), 6-BA was found to have the greatest effect on duckweed biomass and, along with ABA, was most effective in enhancing biomass and starch accumulation (Liu et al., 2019). A previous study also revealed that after waterlogging during anthesis, foliar application of 6-BA significantly heightened the *P*_n_ of wheat leaves and the aboveground biomass and grain yield (Wang X. et al., 2020a). Evidence also suggests that spraying exogenous 6-BA was able to postpone leaf senescence and improve the chlorophyll content and photosynthetic capacity, thereby effectively lessening the harmful effects of flooding in summer maize (Ren et al., 2016). Furthermore, spraying the 6-BA solution before shading during anthesis was also found to delay the senescence of wheat flag leaves under shading treatment, as well as increase dry matter accumulation and reduce yield losses (Li et al., 2019).

At present, research into the mitigating effects of 6-BA application tends to focus on single stress events such as waterlogging or high temperatures, with the research content centering on photosynthetic characteristics and yield. However, few reports have examined the mitigating effects of exogenous 6-BA application on photosynthetic product transport, grain starch, and its starch component content in wheat under the combined stress of WS. This study aimed at ascertaining the effects of spraying several different concentrations of the 6-BA solution on photosynthetic characteristics, photosynthetic product transport, grain starch accumulation, grain filling characteristics, and yield under shading and waterlogging stress after anthesis. Furthermore, the physiological reasons for reducing the damage of WS to wheat yield after spraying the 6-BA solution were analyzed by studying the process of the photosynthetic performance and dry matter accumulation of wheat. This research could provide the theoretical foundation for effective cultivation techniques and measures to alleviate the damage of continuous rainfall after the anthesis of wheat.

## 2 Materials and methods

### 2.1 Experimental design

**Figure 1:**
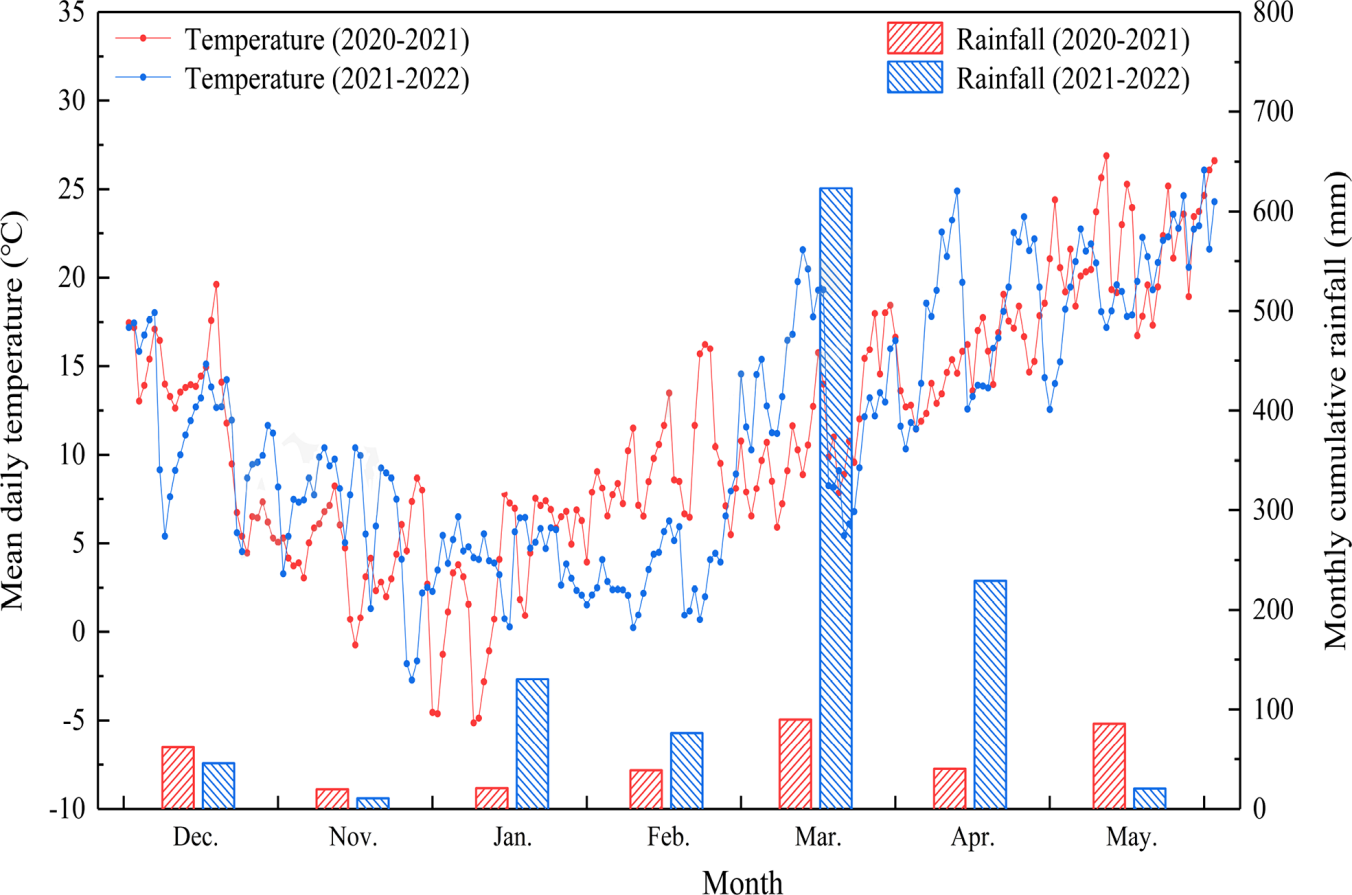
The mean daily temperature and monthly cumulative rainfall during wheat growing seasons (2020–2022).

The experiment was carried out from November 2020 to June 2022 at Anhui Agricultural University Wanzhong Experimental Base, Guohe Town, Lujiang County, Hefei City (117°01′E, 30°57′N). The test site has a subtropical humid monsoon climate, with an average annual temperature of 15°C–16°C, average annual precipitation of 900–1000 mm, an annual sunshine duration of about 2000 h, and an average annual frost-free period of 228 days. The mean daily temperature and monthly cumulative rainfall from 2020 to 2022 during the wheat growing season is shown in **Figure 1**. The waterlogging-tolerant cultivar Yangmai 18 (bred by the Academy of Agricultural Sciences of Lixiahe District, Jiangsu Province) and the waterlogging-sensitive cultivar Sumai 188 (bred by Jiangsu Fengqing Seed Industry Technology Co., Ltd.) were used as test materials. Each box contained 150 kg of paddy soil taken from the 0–20 cm tillage layer. Before sowing in 2020 and 2021, the soil contains 22.3 and 15.8 g·kg^−1^ of organic matter, 1.4 and 0.8 g·kg^−1^ of total nitrogen, 106.5 and 104.4 mg·kg^−1^ of alkali-hydrolyzable nitrogen, 105.0 and 91.0 mg·kg^−1^ of available potassium, and 21.2 and 22.7 mg·kg^−1^ of available phosphorus, respectively. Plants were grown in boxes measuring 70 cm in length, 50 cm wide, and 43 cm high, with a volume of 120 L. Each box contained 150 kg of soil. Round drainage holes with a diameter of 2 cm were arranged around the sides of each box approximately 8 cm from the bottom, with eight along each long side and six on each end. During WS treatment, the drainage holes were plugged with rubber stoppers, while at all other times, they were kept open to ensure sufficient water permeability. The wheat plants were sown on 9 November 2020 and 2 November 2021, five rows per box at a row spacing of 15 cm. Before sowing, each box was supplied with 105 g organic fertilizer, 2.6 g phosphate fertilizer (P_2_O_5_), 5.2 g potassium fertilizer (K_2_SO_4_), and 15.98 g nitrogen fertilizer (46% urea), followed by 6.85 g nitrogen fertilizer as topdressing during the jointing stage. All other field management ways were the same as high-yield cultivation measures in the field.

**Figure 2:**
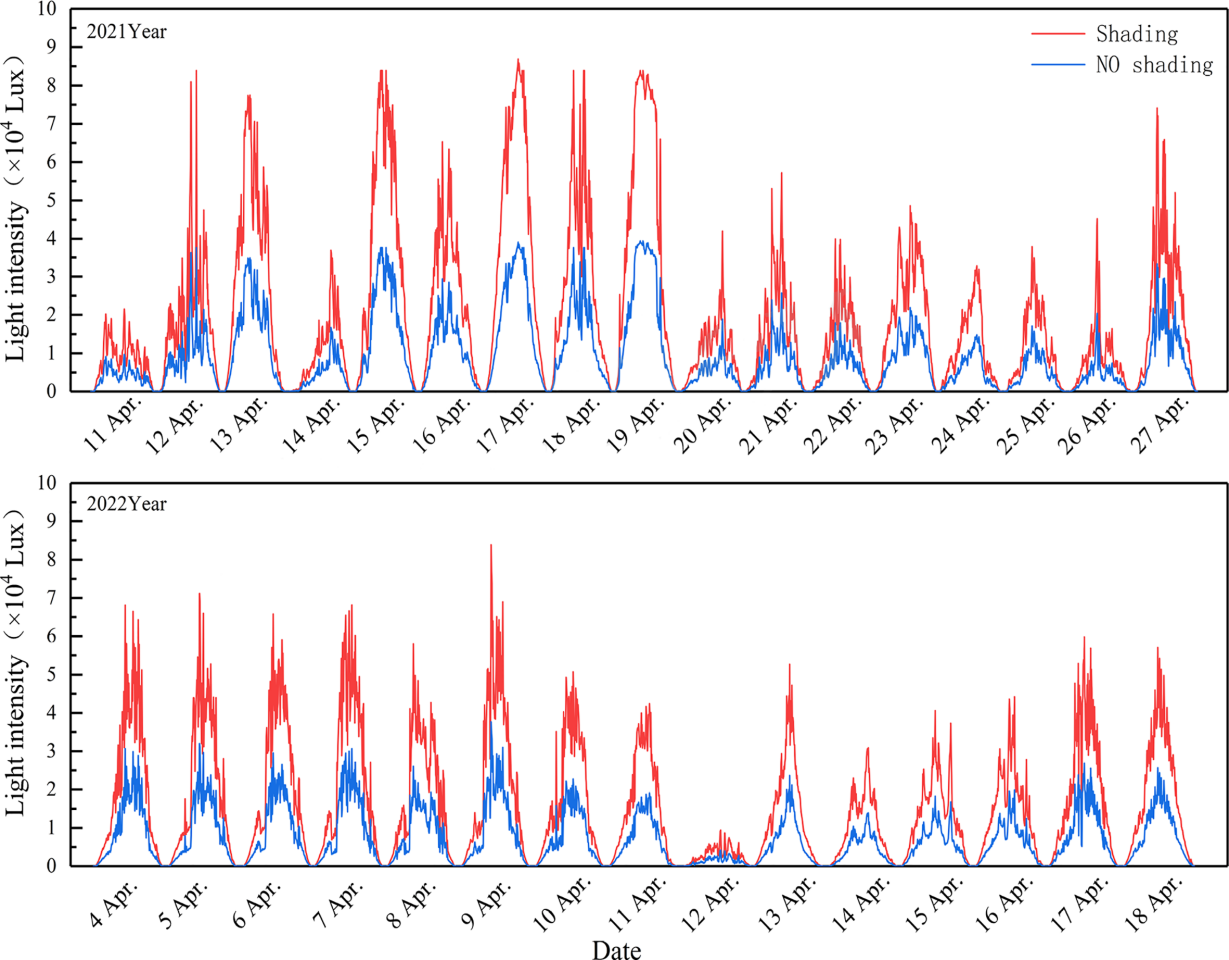
Changes of light intensity under shading treatment.

WS were carried out during anthesis at durations of 7, 11, and 15 days. Shading and waterlogging treatment were carried out simultaneously, with shade nets used to create a shading rate of approximately 45%. The light intensity after shading is shown in **Figure 2**. Yangmai 18 underwent WS treatment at 19:00 on 10 April 2021 and 4 April 2022, while Sumai 188 began treatment at 19:00 on 12 April 2021 and 4 April 2022. The shade net was fixed to an open arched structure, the top of which was 1.8 m above the ground to allow sufficient ventilation. During waterlogging, the drainage holes were plugged to allow approximately 2 cm of water to accumulate on the soil surface. Rainproof measures were also taken during WS to mitigate the effect of external rainfall events. At 9:00 a.m. the day following WS treatment, plants were sprayed with one of three concentrations of the 6-BA solution (15, 25, and 35 mg·L^−1^). As a control, the same amount of distilled water was used. The spraying amount was 500 ml for each box, so as to ensure that each leaf was evenly covered with the reagent. Each treatment set consisted of 12 boxes.

### 2.2 Acquisition of samples

Single stems that bloomed on the same day and had similar-sized wheat ears were marked in each treatment to indicate anthesis. Following WS treatment, and starting from 10 days after anthesis, 20–25 of the marked wheat ears were then randomly selected from each treatment every 5 days (see **Table 1** for sampling times). The grains were then collected and the remaining kernels were removed after heating at 105°C for 15 min. These samples were kiln-dried to a steady weight at 80°C for the analysis of starch and each starch component, and for grain dry matter accumulation.

**Table 1:** Sampling time for different treatments under waterlogging and shading after anthesis.

Treatment	Sample time					
WS_7_	10 DAA	15 DAA	20 DAA	25 DAA	30 DAA	35 DAA
WS_11_	*—*	15 DAA	20 DAA	25 DAA	30 DAA	35 DAA
WS_15_	*—*	*—*	20 DAA	25 DAA	30 DAA	35 DAA

WS_7_, WS_11_, and WS_15_ respectively represent treatments under waterlogging and shading for 7, 11, and 15 days after anthesis. Shade nets with a shading rate of about 45% were used for shading, and waterlogging preserves about 2 cm of moisture on the surface of the soil. DAA represents days after anthesis. — represents not sampling.

### 2.3 Measurements

#### 2.3.1 Morphology of grains and starch granules

Grain morphology was observed in samples obtained as described above and in **Table 1**, every 5 days from 10 days after anthesis. One to two seeds from the bottom of the intermediate spikelet were selected for observations using a SZX16 stereo microscope (OLYMPUS, Japan).

For observations of starch grain morphology, one to two wheat ears were randomly selected from the marked wheat plant after reaching maturity. One to two grains at the bottom of the middle spikelet were then selected and cut along the middle with a blade to provide cross-sections for observations of the endosperm. The samples were fixed on a copper column with double-sided carbon glue then plated with gold using an ion sputtering device then the ultrastructure of the starch granules was observed using an S-4800 scanning electron microscope (HITACHI, Japan).

#### 2.3.2 Photosynthetic parameters of flag leaves

The LI-6400XT portable photosynthetic system (LI-COR Co., USA), with an open gas circuit, was used to set the rate of photosynthetically active radiation (PAR) to 1200 μmol·m^−2^·s^−1^. In each treatment, three flag leaves of wheat with the same growth and development process were measured from 9:00 a.m. to 11:00 a.m. When the net photosynthetic rate of wheat tended to be stable, the photosynthetic parameters at this time were recorded. Each flag leaf was measured three times then the average value was determined. Measurements were performed every 5 days from 10 to 25 days after anthesis.

#### 2.3.3 Isotope δ^13^C analysis

After spraying with the 6-BA solution the day after WS, 10 flag leaves were randomly selected from the marked single stem of wheat from each treatment; then the flag leaves were then sealed with transparent PVC plastic bags, injected with 5 ml of ^13^CO_2_ gas into the plastic bags using a medical syringe, and assimilated for 60 min under natural light conditions at 10:00–11:00 a.m. on the same day. Three flag leaves were then removed, fixed at 105°C for 15 min, and kiln-dried at 80°C. After reaching maturation, three unsampled wheat ears were selected, the grains removed, fixed, and dried. They were then ground to a powder using an A11 basic analytical mill (IKA, Germany) and passed through a 100-mesh screen. The Sumai 188 after 11 days under WS was selected for analysis of δ^13^C using flag leaves and grains were treated with 25 mg·L^−1^ of the 6-BA solution. To do so, a Sercon Integra 2 Elemental Analysis-Stable Isotope Ratio Mass Spectrometer (EA-IRMS) was used with the ^13^CO_2_-labeled flag leaves and grains (SerCon, Britain).

δ^13^C_TOC_ values were obtained using the PDB international standard as a reference, as follows:


(1) δ13CTOC(‰)=[R(C 13/C 12sample )R(C 13/C 12VPDB )]−1×1000

where R (^13^C/^12^C_VPDB_) is the carbon isotope abundance ratio of the international standard Vienna Pee Dee Belemnite (VPDB). The analytical precision of the δ^13^C_TOC_ values was set at ± 0.2‰.

#### 2.3.4 Grain starch and its component content

The total starch content of the grains was determined by anthrone colorimetry (Jia and Zhang, 2008). After drying, the wheat kernels were ground and then passed through a 100-mesh sieve. Next, 8 ml of 80% ethanol was added to about 0.1 g (accurate to 1mg) of the sample, heated in a water bath at 80°C for 30 min, cooled, and then centrifuged at 5000×*g* for 15 min. The supernatant was then discarded, 2 ml of distilled water was added to the precipitate, shaken, and then boiled for 20 min before cooling and adding 2 ml of 9.2 mol·L^−1^ HCl_4_. The samples were then shaken for a further 10 min before adding 6 ml of distilled water and centrifuging at 5000×*g* for 15 min. The resulting supernatant was then poured into a 50 ml volumetric flask. The process was repeated three times, giving a total sample volume of 50 ml, respectively. Next, 0.1 ml of the extract solution was added to 4 ml of 0.2% anthrone, boiled for 15 min, cooled, and observed using a spectrophotometer at a wavelength of 620 nm (Agilent Cary 300, USA). The starch standard curve was then used to calculate the starch content of each sample.

The amylose content was determined using a coupled spectrophotometer (Zhang et al., 2009). After drying, the wheat grains were ground into a powder, passed through a 100-mesh sieve, and then degreased with ether. Next, 10 ml of 0.5 mol·L^−1^ KOH solution was added to about 0.1 g (accurate to 1 mg) of defatted sample, boiled for 10 min, then diluted to 50 ml with distilled water. Any foam was removed with ethanol then the sample was left to stand for 20 min. Next, 30 ml of distilled water was added to 2.5 ml of solution; the pH was adjusted to 3.5 using 0.1 mol·L^−1^ HCl, and 0.5 ml iodine reagent was added (diluted 2.0 g potassium iodide and 0.2 g iodine with distilled water to 100 ml). A blank solution without an iodine reagent was diluted to 50 ml then left to stand for 20 min for use as a control. Colorimetry was then performed using a spectrophotometer at wavelengths of 630 and 460 nm (Agilent Cary 300, USA). The amylose content was obtained using the amylose standard curve then the amylopectin content was determined as follows:

Amylopectin content (%) = total starch content (%)−amylose content (%).

#### 2.3.5 Grain filling measurements

Every 5 days from 10 days after anthesis, 15–20 wheat ears showing consistent growth and development were sampled. The grains were removed then fixed at 105°C for 15 min, dried at 80°C to a constant weight, weighted, and then converted into the 1000-grain weight. The Logistic equation *Y* = K/[1+exp(A+B*t*)] (Darroch and Baker, 1990) was used to fit the variation in 1000-grain weight with the number of days after anthesis, where *Y* is the 1000-grain weight of the observed grain (g), *t* is the number of days (d) from anthesis until observation, A and B are determined parameters, and K is the fitted maximum 1000-grain weight (g). Least-squares estimates of K, A, and B were determined by nonlinear regression. The first and second derivatives of this Logistic equation are obtained, and the following parameters were then determined:

Effective days of grain filling (*D*):


(2) $$D=\frac{\ln(1/9)-A}{B}$$

Duration of the gradual grain filling period (*D*_1_):


(3) $$D_{1}=\frac{A-1.317}{B}$$

Duration of the rapid grain filling period (*D*_2_):


(4) $$D_{2}=\frac{A-1.317}{B}-\frac{A+1.317}{B}$$

Duration of the slow grain filling period (*D*_3_):


(5) $$D_{3}=D-D_{1}-D_{2}$$

Average grain-filling rate (*V*_mean_):


(6) $$V_{mean}=K/D$$

Maximum grain-filling rate (*V*_max_):


(7) $$V_{max}=-KB/4$$

Time point of the maximum grain-filling rate (*T*_max_):


(8) $$T_{max}=-A/B$$

#### 2.3.6 Yield and contributing factors

After reaching maturity, three boxes of unsampled wheat were selected from each treatment for the analysis of spikes, kernel numbers per spike, thousand-grain weight, and grain yield.

### 2.4 Data processing and analysis

Data were analyzed using the SPSS 22.0 software to fit the logistic curve with related parameters of starch, each starch component, and grain dry matter data. Duncan’s method was used to perform multiple comparisons of the measured data. Origin 2017 was used for graphing and Photoshop was used for image annotation and processing.

## 3 Results

### 3.1 Effects of spraying 6-BA after WS on the morphology of grain and starch granules

#### 3.1.1 Grain morphology

**Figure 3:**
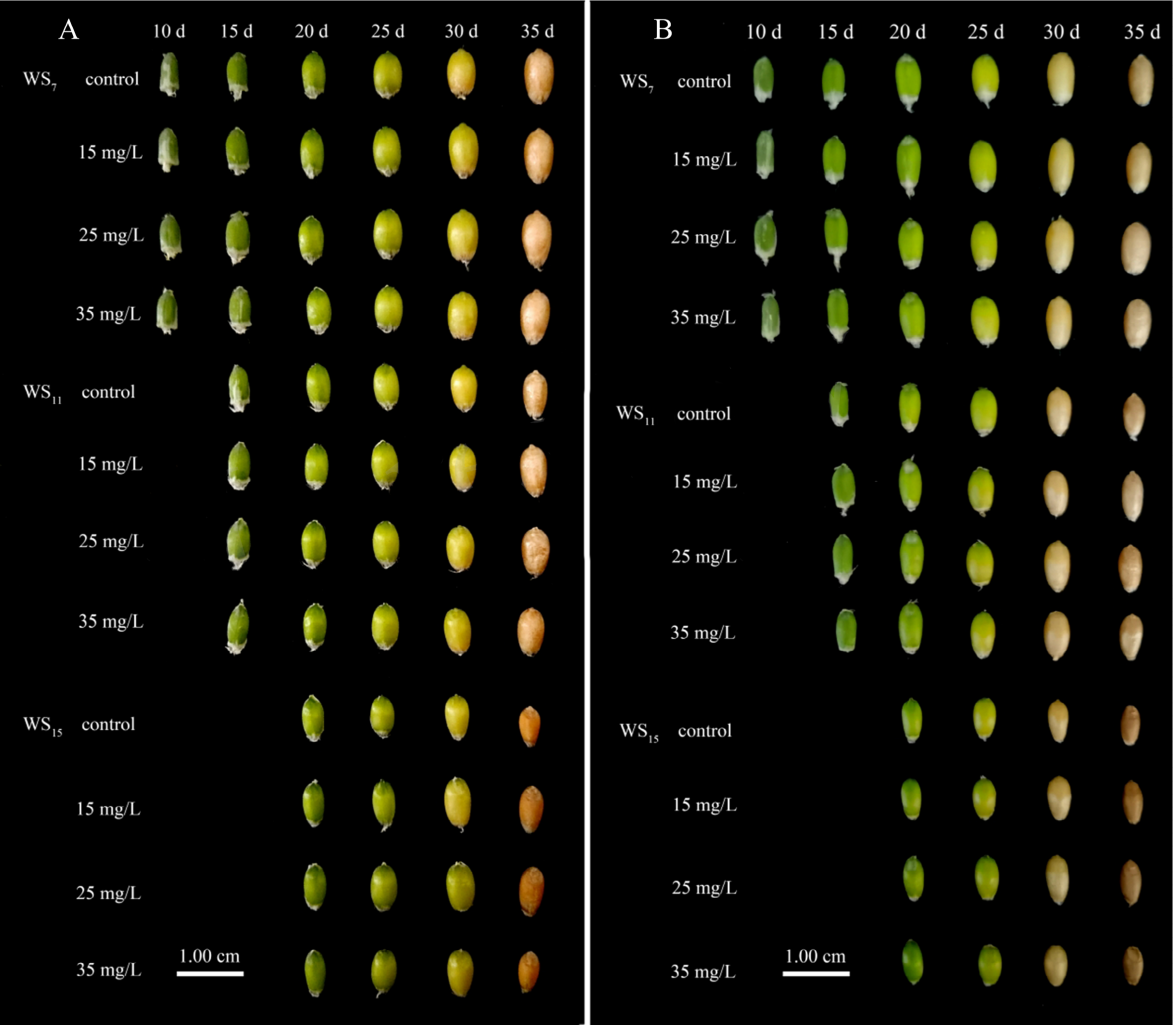
The effect of exogenous 6-BA on grain morphology of wheat under waterlogging and shading. The waterlogging-tolerant (Yangmai 18, 2021 **A**) and the waterlogging-sensitive (Sumai 188, 2021 **B**) were waterlogging and shading during anthesis. WS_7_, WS_11_, and WS_15_ respectively represent treatments under waterlogging and shading for 7, 11, and 15 days after anthesis. Shade nets with a shading rate of about 45% were used for shading, and waterlogging preserves about 2 cm of moisture on the surface of the soil. Control is the treatment of spraying the same amount of distilled water; 15 mg·L^−1^, 25 mg·L^−1^, and 35 mg·L^−1^ are the concentrations of 6-BA sprayed.

The degree of grain filling gradually deteriorated with increasing WS; however, the effect was alleviated after the application of 6-BA. Under each duration of WS, grain plumpness increased after the application of 6-BA, and the difference in grain fullness was obvious between the treatment and control groups. The grain ripening process was faster in the waterlogging-sensitive variety Sumai 188 (**Figure 3B**) than the waterlogging-tolerant Yangmai 18 (**Figure 3A**), while the degree of overall grain fullness was greater in Yangmai 18 than Sumai 188.

#### 3.1.2 Morphology of starch granules

**Figure 4:**
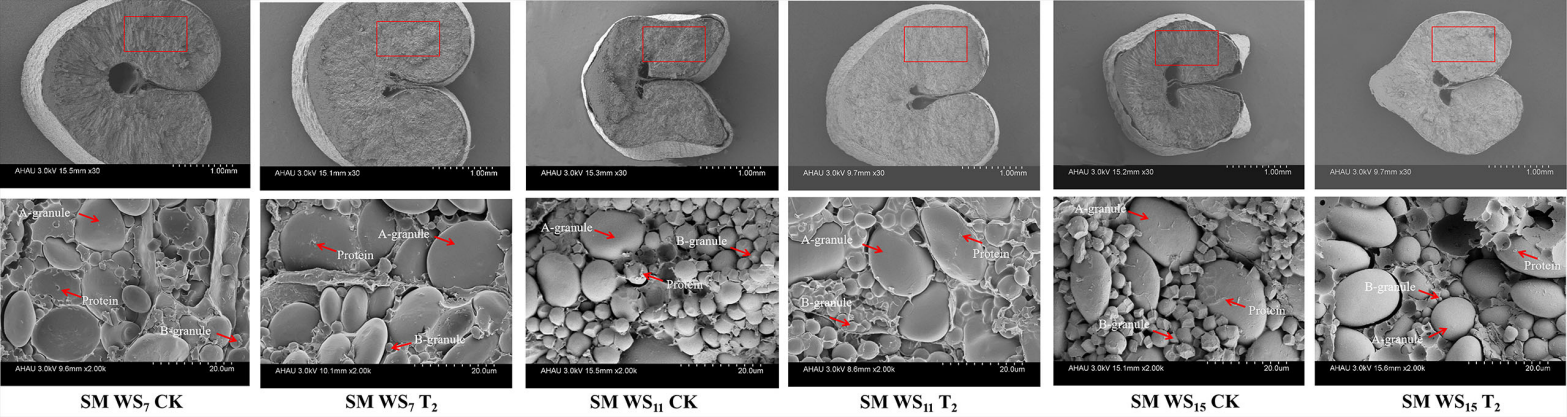
The effects of exogenous 6-BA on final starch grain morphology under waterlogging and shading. SM, Sumai 188, 2021. WS_7_, WS_11_, and WS_15_ respectively represent treatments under waterlogging and shading for 7, 11, and 15 days after anthesis. Shade nets with a shading rate of about 45% were used for shading, and waterlogging preserves about 2 cm of moisture on the surface of the soil. CK refers to the control sprayed with distilled water; T_2_: the concentration of 6-BA sprayed is 25 mg·L^−1^. The red rectangles in the graphs indicate the range of areas to be observed.

The ultrastructure of the wheat grains at maturity was observed in Sumai 188 using a scanning electron microscope (**Figure 4**). As shown, A- and B-type starch granules in the grain were closely arranged and filled the endosperm cells, with granular proteins in between. Meanwhile, with increasing WS, the cross-sectional area of the grains decreased; moreover, these grains became shrunken and deformed. In terms of starch granule morphology, the longer the duration of WS, the smaller the individual volume of A-type starch granules and the greater the number of deformed B-type granules. After WS for 11 days, some irregular B-type starch granules were observed, while after 15 days, most B-type granules formed irregular polygons. Compared with the control, the volume of A-type starch granules in the grains increased significantly after the application of 6-BA, and deformation of the B-type granules decreased.

### 3.2 Effects of spraying 6-BA after WS on photosynthetic parameters of the flag leaves

**Figure 5:**
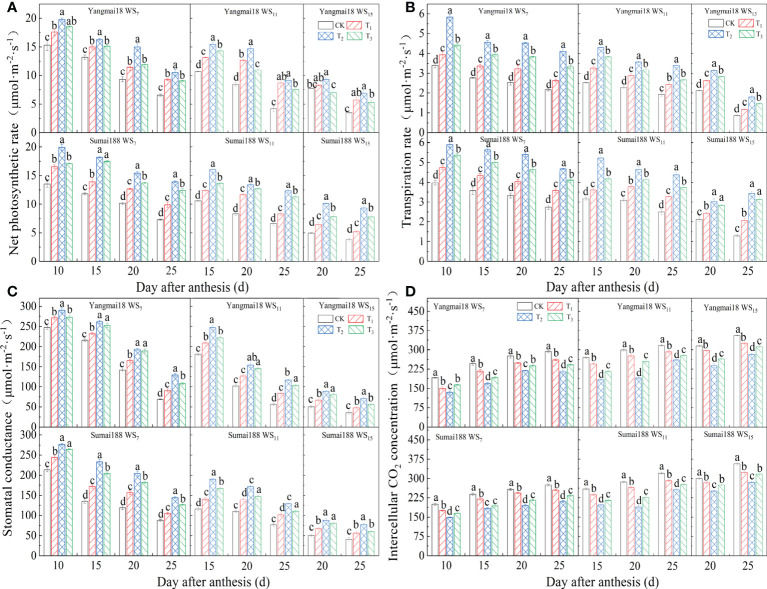
The effect of exogenous 6-BA on Pn **(A)**, Tr **(B)**, Gs **(C)**, Ci **(D)** of wheat under waterlogging and shading in 2021. WS_7_, WS_11_, and WS_15_ respectively represent treatments under waterlogging and shading for 7, 11, and 15 days after anthesis. Shade nets with a shading rate of about 45% were used for shading, and waterlogging preserves about 2 cm of moisture on the surface of the soil. CK refers to the control sprayed with distilled water. T_1_, T_2_, and T_3_ refer to the concentrations of 6-BA sprayed (15, 25, and 35 mg·L^−1^). Each value is expressed as mean ± SE (*n* = 3). Different lowercase letters embedded at the top of the histogram express significant differences between treatments (*p<* 0.05).

As shown in **Figure 5A**, the *P*_n_ of the flag leaves continued to decrease with growth after anthesis, and this decrease increased with increased WS. The *P*_n_ of the flag leaves increased significantly after the application of the 6-BA solution (*p<* 0.05), and was highest at a concentration of 25 mg·L^−1^ of 6-BA, with significant differences compared with the remaining two treatments (*p<* 0.05). Taking Sumai 188 after 20 days after anthesis as an example, the *P*_n_ of the flag leaves increased by 19.74%, 34.13%, and 25.97% compared with the control under WS treatment for 7 days after spraying 15, 25, and 35 mg·L^−1^ of the 6-BA solution, respectively. Meanwhile, after 11 days under WS, increases of 28.57%, 37.66%, and 34.38% were observed, while after 15 days, increases of 22.80%, 50.83%, and 36.60% were noted, respectively.

As shown in **Figures 5B, C** the changes in *G*_s_ and *T*_r_ in the control flag leaves after anthesis were consistent with those of *P*_n_, with a continual decrease with growth, and WS. In contrast, compared with the control, both *G*_s_ and *T*_r_ increased significantly after the application of 6-BA (*p<* 0.05). Meanwhile, as shown in **Figure 5D**, the *C*_i_ of the control flag leaves after anthesis increased gradually with growth and WS. In comparison, the application of 6-BA caused a significant decrease in the *C*_i_ of the flag leaves (*p<* 0.05).

### 3.3 Effect of spraying 6-BA after WS on the δ^13^C of the flag leaves and mature grains

**Figure 6:**
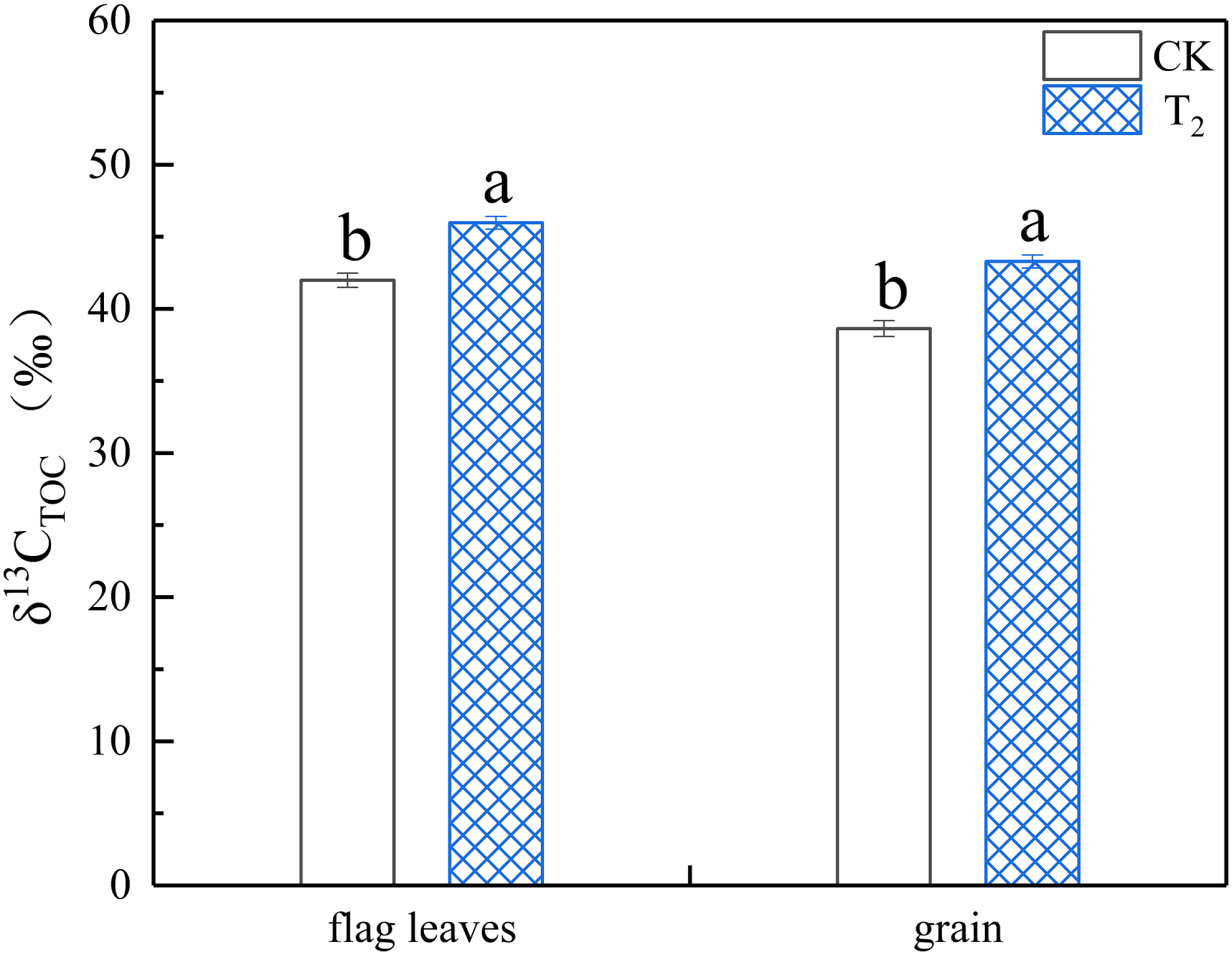
The effect of exogenous 6-BA on flag leaves and grain δ^13^C of wheat under waterlogging and shading treatment of 11 days in 2021. CK refers to the control sprayed with distilled water. T_2_: the concentration of 6-BA sprayed is 25 mg·L^−1^. Shade nets with a shading rate of about 45% were used for shading, and waterlogging preserves about 2 cm of moisture on the surface of the soil. Each value is expressed as mean ± SE (*n* = 3). Different lowercase letters embedded at the top of the histogram express significant differences among treatments (*p<* 0.05).

As shown in **Figure 6**, the δ^13^C of the flag leaves increased significantly by 8.67% compared with the control after spraying 25 mg·L^−1^ of the 6-BA solution for 24 h, while that of the mature grains increased significantly by 10.77% (*p<* 0.05). The transport rate of δ^13^C from the flag leaves to the grains was 92.02% in the control group, while that spraying 25 mg·L^−1^ of the 6-BA solution was 94.18%. Overall, after application of each concentration of 6-BA, the transport of assimilates from the flag leaves to the grains was significantly improved.

### 3.4 Effect of spraying 6-BA after WS on the content of grain starch and its component

#### 3.4.1 Starch content

**Figure 7:**
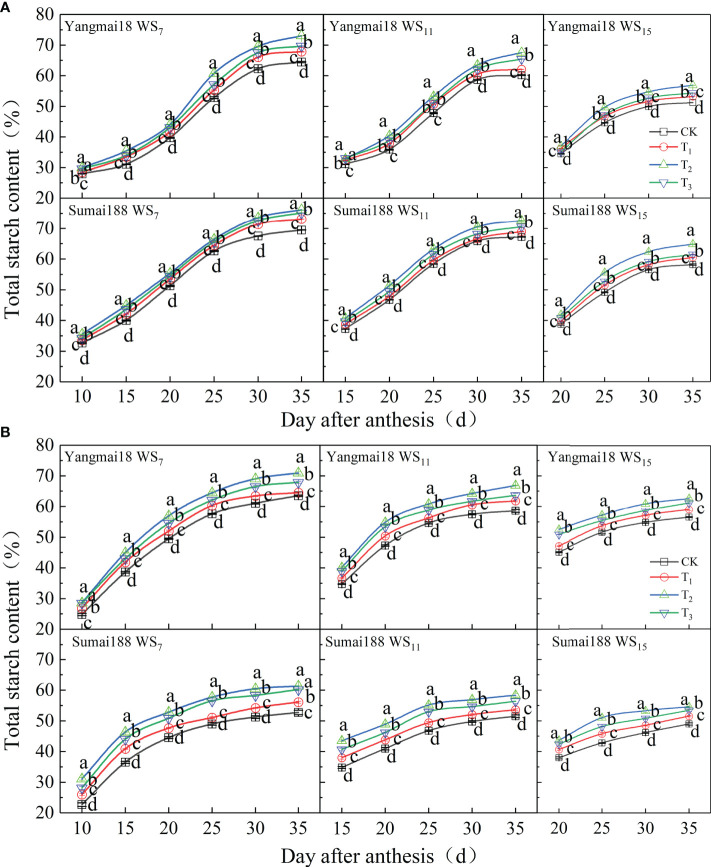
The effect of exogenous 6-BA on total starch accumulation of wheat under waterlogging and shading in 2021 **(A)** and 2022 **(B)**. WS_7_, WS_11_, and WS_15_ respectively represent treatments under waterlogging and shading for 7, 11, and 15 days after anthesis. Shade nets with a shading rate of about 45% were used for shading, and waterlogging preserves about 2 cm of moisture on the surface of the soil. CK refers to the control sprayed with distilled water. T_1_, T_2_, and T_3_ refer to the concentration of 6-BA sprayed (15, 25, and 35 mg·L^−1^). Each value is expressed as mean ± SE (*n* = 3). Different lowercase letters embedded in the linear graph express significant differences among treatments (*p<* 0.05).

As shown in **Figure 7**, the growth of the grain starch showed a rapid increase from 15 to 25 days after anthesis followed by gradual stabilization from 25 to 35 days. The longer the duration of WS, the lower the final accumulation of starch; a significant increase was observed following the application of 6-BA during the entire grain-filling process compared with the control (**Figures 7A, B**, *p<* 0.05). Moreover, starch accumulation was significantly higher after spraying 25 mg·L^−1^ of the 6-BA solution compared with the remaining two concentrations. Take the 2021 year results as an example, after WS for 15 days, the final accumulation of starch in the Sumai 188 grains increased by 3.85%, 10.24%, and 5.28% compared with the control after spraying 15, 25, and 35 mg·L^−1^ of the 6-BA solution, respectively.

#### 3.4.2 Amylose and amylopectin content

As shown in **Tables 2**, **3**, the amylose and amylopectin content gradually increased with wheat grain development; however, the longer the duration of WS, the lower the final accumulation. The different cultivars, treatment durations of WS, and spraying concentrations of 6-BA significantly affected the final amylose and amylopectin contents from wheat grains. Under the same treatment duration of WS, except at 10 days after anthesis, the content of amylose and amylopectin was improved remarkably following the application of 6-BA in contrast with the control treatment during the grouting period (*p<* 0.05), suggesting that 6-BA improved the accumulation of amylose and amylopectin in the wheat grains. As an example, after WS for 15 days, the final amylose content of the Sumai 188 grains increased by 3.44%, 6.60%, and 3.15% compared with the control after spraying 15, 25, and 35 mg·L^−1^ of the 6-BA solution, while the final accumulation of amylopectin increased by 3.95%, 11.15%, and 5.82%, respectively.

**Table 2:** The effect of exogenous 6-BA on amylose accumulation of wheat under waterlogging and shading in 2021.

Cultivar	Treatment		Amylose content (%)					
			10DAA	15DAA	20DAA	25DAA	30DAA	35DAA
Yangmai 18	WS_7_	CK	1.14a ± 0.06	2.46c ± 0.06	4.03c ± 0.04	7.76d ± 0.06	11.93c ± 0.14	13.50c ± 0.09
		T_1_	1.20a ± 0.05	2.83b ± 0.09	4.30c ± 0.05	8.30c ± 0.03	12.94b ± 0.11	14.00bc ± 0.08
		T_2_	1.22a ± 0.12	3.25a ± 0.06	5.16a ± 0.17	9.34a ± 0.05	14.21a ± 0.35	15.47a ± 0.33
		T_3_	1.17a ± 0.05	3.01ab ± 0.08	4.67b ± 0.07	8.56b ± 0.10	12.96b ± 0.30	14.30b ± 0.07
	WS_11_	CK	—	2.13c ± 0.08	3.50d ± 0.11	6.46d ± 0.12	11.12c ± 0.07	12.11d ± 0.07
		T_1_	—	2.47b ± 0.04	4.02c ± 0.08	7.30c ± 0.17	11.93b ± 0.06	12.76c ± 0.09
		T_2_	—	2.83a ± 0.06	4.85a ± 0.03	8.47a ± 0.11	13.07a ± 0.07	14.43a ± 0.11
		T_3_	—	2.64b ± 0.04	4.36b ± 0.09	7.91b ± 0.10	12.16b ± 0.09	13.60b ± 0.02
	WS_15_	CK	—	—	3.01d ± 0.07	6.06d ± 0.09	10.61d ± 0.06	11.45c ± 0.08
		T_1_	—	—	3.65c ± 0.08	7.07c ± 0.10	11.23c ± 0.10	11.99b ± 0.10
		T_2_	—	—	4.57a ± 0.11	8.02a ± 0.10	12.28a ± 0.04	13.11a ± 0.09
		T_3_	—	—	3.92b ± 0.06	7.50b ± 0.05	11.69b ± 0.07	12.21b ± 0.09

Sumai 188	WS_7_	CK	1.22a ± 0.09	2.78c ± 0.10	6.31d ± 0.07	10.95d ± 0.08	13.49d ± 0.06	13.98c ± 0.09
		T_1_	1.15a ± 0.03	3.21b ± 0.04	6.61c ± 0.05	11.34c ± 0.04	14.11c ± 0.06	14.84b ± 0.06
		T_2_	1.29a ± 0.05	3.80a ± 0.04	7.37a ± 0.05	12.24a ± 0.14	15.09a ± 0.04	15.49a ± 0.08
		T_3_	1.20a ± 0.03	3.41b ± 0.06	6.93b ± 0.04	11.64b ± 0.07	14.51b ± 0.09	14.95b ± 0.06
	WS_11_	CK	—	2.61d ± 0.06	5.89d ± 0.07	10.33d ± 0.05	13.22d ± 0.03	13.52d ± 0.09
		T_1_	—	2.96c ± 0.04	6.25c ± 0.03	11.30b ± 0.06	13.42c ± 0.03	13.88c ± 0.02
		T_2_	—	3.40a ± 0.07	6.83a ± 0.04	11.85a ± 0.04	14.47a ± 0.05	14.77a ± 0.02
		T_3_	—	3.14b ± 0.01	6.57b ± 0.04	10.87c ± 0.05	13.95b ± 0.04	14.25b ± 0.07
	WS_15_	CK	—	—	5.58d ± 0.05	8.26c ± 0.12	11.55c ± 0.07	12.06d ± 0.03
		T_1_	—	—	5.82c ± 0.03	8.69b ± 0.08	12.03b ± 0.08	12.49c ± 0.07
		T_2_	—	—	6.79a ± 0.02	9.49a ± 0.11	12.45a ± 0.05	13.31a ± 0.03
		T_3_	—	—	6.36b ± 0.03	9.19a ± 0.06	12.07b ± 0.02	12.83b ± 0.08

Significant level			10DAA	15DAA	20DAA	25DAA	30DAA	35DAA
Cultivar (C)			ns	**	**	**	**	**
Treatment duration (D)			—	**	**	**	**	**
Concentrations of 6-BA (T)			ns	**	**	**	**	**
C×D			—	**	**	**	**	**
C×T			ns	**	ns	**	*	**
D×T			—	**	ns	**	**	**
C×D×T			—	ns	ns	**	*	**

WS_7_, WS_11,_ and WS_15_ respectively represent treatments under waterlogging and shading for 7, 11, and 15 days after anthesis. Shade nets with a shading rate of about 45% were used for shading, and waterlogging preserves about 2 cm of moisture on the surface of the soil. CK refers to the control sprayed with distilled water, T_1_, T_2_, T_3_ refer to 6-BA sprayed at a concentration of 15, 25, and 35 mg·L^−1^, respectively. DAA represents days after anthesis. — represents not sampling. Each value is expressed as mean ± SE (n = 3). Different letters labeled after the value among the treatments with different concentrations of 6-BA refer to significant differences at p< 0.05. ** represents significant differences at the 1% level. * represents significant differences at the 5% level. ns represents no significant difference.

**Table 3:** The effect of exogenous 6-BA on amylopectin accumulation of wheat under waterlogging and shading in 2021.

Cultivar	Treatment		Amylopectin content (%)					
			10DAA	15DAA	20DAA	25DAA	30DAA	35DAA
Yangma 18	WS_7_	CK	13.07a ± 0.33	28.39b ± 0.99	35.59c ± 0.67	45.22d ± 0.27	50.29c ± 0.48	50.92c ± 0.23
		T_1_	13.25a ± 0.48	30.43ab ± 0.27	37.40b ± 0.45	47.18c ± 0.12	52.42b ± 0.77	53.85b ± 0.15
		T_2_	13.96a ± 0.34	32.26a ± 0.72	40.58a ± 0.22	51.62a ± 0.05	55.99a ± 0.49	57.87a ± 0.65
		T_3_	13.88a ± 0.36	31.04a ± 0.71	38.53b ± 0.35	48.73b ± 0.30	54.51a ± 0.35	55.04b ± 0.28
	WS_11_	CK	—	22.41c ± 0.38	32.58c ± 0.42	42.76d ± 0.29	32.58c ± 0.42	48.84d ± 0.42
		T_1_	—	24.35b ± 0.27	34.31bc ± 0.60	44.88c ± 0.40	34.31bc ± 0.60	50.77c ± 0.59
		T_2_	—	26.09a ± 0.16	37.50a ± 0.37	48.65a ± 0.50	37.50a ± 0.37	55.08a ± 0.33
		T_3_	—	24.46b ± 0.59	35.02b ± 0.72	46.37b ± 0.41	35.02b ± 0.72	53.28b ± 0.14
	WS_15_	CK	—	—	28.18c ± 0.52	38.82d ± 0.40	42.71d ± 0.33	44.91c ± 0.24
		T_1_	—	—	31.05b ± 0.61	40.19c ± 0.37	46.67c ± 0.40	47.92b ± 0.40
		T_2_	—	—	33.48a ± 0.09	44.96a ± 0.38	51.25a ± 0.56	51.39a ± 0.51
		T_3_	—	—	31.20b ± 0.08	42.15b ± 0.45	48.64b ± 0.56	49.02b ± 0.46

Sumai 188	WS_7_	CK	14.37c ± 0.21	27.84d ± 0.35	42.59c ± 0.50	50.23d ± 0.34	53.52c ± 0.47	53.33d ± 0.35
		T_1_	15.18bc ± 0.20	29.81c ± 0.29	44.07bc ± 0.83	52.66c ± 0.47	56.78b ± 0.60	56.79c ± 0.39
		T_2_	16.14a ± 0.36	33.44a ± 0.29	46.37a ± 0.52	55.12a ± 0.14	58.47a ± 0.15	60.55a ± 0.13
		T_3_	15.61ab ± 0.24	32.08b ± 0.18	44.94ab ± 0.47	54.04b ± 0.25	57.52ab ± 0.38	59.33b ± 0.39
	WS_11_	CK	—	26.70c ± 0.31	39.73d ± 0.23	46.90b ± 0.34	51.15c ± 0.24	50.15d ± 0.23
		T_1_	—	28.73b ± 0.41	41.49c ± 0.37	47.65b ± 0.15	52.22bc ± 0.48	54.15c ± 0.28
		T_2_	—	30.53a ± 0.26	44.38a ± 0.35	52.00a ± 0.33	56.48a ± 0.42	59.00a ± 0.17
		T_3_	—	28.91b ± 0.18	42.76b ± 0.28	50.04a ± 0.52	53.32b ± 0.23	55.33b ± 0.22
	WS_15_	CK	—	—	33.01d ± 0.37	40.76c ± 0.36	45.25d ± 0.36	43.87d ± 0.20
		T_1_	—	—	34.04c ± 0.18	42.66b ± 0.36	46.94c ± 0.33	48.30c ± 0.25
		T_2_	—	—	37.34a ± 0.29	45.98a ± 0.15	51.43a ± 0.44	51.82a ± 0.35
		T_3_	—	—	35.82b ± 0.27	43.34b ± 0.23	49.15b ± 0.29	49.71b ± 0.40

Significant level			10DAA	15DAA	20DAA	25DAA	30DAA	35DAA
Cultivar (C)			**	**	**	**	**	**
Treatment duration (D)			—	**	**	**	**	**
Concentrations of 6-BA (T)			**	**	**	**	**	**
C×D			—	**	**	**	**	**
C×T			*	**	**	**	ns	**
D×T			—	**	**	**	**	**
C×D×T			—	**	**	**	**	**

WS_7_, WS_11_, and WS_15_ respectively represent treatments under waterlogging and shading for 7, 11, and 15 days after anthesis. Shade nets with a shading rate of about 45% were used for shading, and waterlogging preserves about 2 cm of moisture on the surface of the soil. CK refers to the control sprayed with distilled water, T_1_, T_2_, and T_3_ refer to 6-BA sprayed at a concentration of 15, 25, and 35 mg·L^−1^, respectively. DAA represents days after anthesis. — represents not sampling. Each value is expressed as mean ± SE (n = 3). Different letters labeled after the value among the treatments with different concentrations of 6-BA refer to significant differences at p< 0.05. ** represents significant differences at the 1% level. * represents significant differences at the 5% level. ns represents no significant difference.

### 3.5 Effect of spraying 6-BA after WS on grain filling

A logistic equation served to fit the grain-filling, with the coefficient factor of each equation reaching a notable level (**Table 4**). The maximum theoretical 1000-kernel weight was higher after the application of 6-BA compared with the control, and the number of effective days of grain filling (*D*) was prolonged in both cultivars (except in Yangmai 18 after WS for 15 days and Sumai 188 after WS for 11 days in 2021, and after WS for 7 days during in 2022). Moreover, under the same treatment duration of WS, the average grain filling rate (*V*_mean_) increased significantly after spraying the 6-BA solution, and the longer the treatment duration of WS, the lower the average grain filling rate. Compared with the control, spraying the 6-BA solution increased the duration of gradual grain filling in Yangmai 18 following 7 days of WS in 2021, while the rapid and slow grain filling periods increased following 11 and 15 days of WS in 2021 and 2022. Of the three concentrations of 6-BA, spraying 25 mg·L^−1^ of the 6-BA solution had the greatest effect. Take the 2021 results as an example; in Yangmai 18, spraying 25 mg·L^−1^ of the 6-BA solution following 7, 11, and 15 days of WS resulted in an increase in *V*_mean_ by 6.27%, 5.59%, and 21.20% compared with the control, while in Sumai 188, increases of 7.84%, 5.08%, and 15.11% were observed, respectively.

**Table 4:** The effect of exogenous 6-BA on the grout characteristic of wheat grain under waterlogging and shading.

Year	Cultivar	Treatment		Model	R^2^	*D*	*D*_1_	*D*_2_	*D*_3_	*V*_mean_	*V*_max_	*T*_max_
						(d)	(d)	(d)	(d)	(g·d^−1^)	(g·d^−1^)	(d)
2021	Yangmai 18	WS_7_	CK	*Y*=40.8284/(1+exp(4.0300-0.209079*t*))	0.9959	29.7841	12.9760	12.5981	4.2100	1.3708	2.1341	19.2750
			T_1_	*Y*=42.1515/(1+exp(3.8009-0.198696*t*))	0.9970	30.1874	12.5010	13.2564	4.4300	1.3963	2.0938	19.1292
			T_2_	*Y*=43.7910/(1+exp(4.2770-0.215386*t*))	0.9936	30.0587	13.7428	12.2292	4.0867	1.4568	2.3580	19.8574
			T_3_	*Y*=43.0673/(1+exp(4.2196-0.209505*t*))	0.9940	30.6285	13.8546	12.5725	4.2014	1.4061	2.2557	20.1408
		WS_11_	CK	*Y*=39.6341/(1+exp(4.5285-0.214369*t*))	0.9951	31.3745	14.9812	12.2872	4.1061	1.2633	2.1241	21.1248
			T_1_	*Y*=40.5208/(1+exp(4.3343-0.207992*t*))	0.9979	31.4028	14.5068	12.6639	4.2320	1.2904	2.1070	20.8388
			T_2_	*Y*=43.2746/(1+exp(3.7295-0.179306*t*))	0.9971	33.0723	13.4622	14.6983	4.9118	1.3085	1.9388	20.8114
			T_3_	*Y*=41.2151/(1+exp(4.0977-0.198904*t*))	0.9961	31.6481	13.9801	13.2426	4.4254	1.3023	2.0495	20.6014
		WS_15_	CK	*Y*=24.6955/(1+exp(6.1919-0.324780*t*))	0.9997	25.8302	15.0099	8.1101	2.7102	0.9561	2.0052	19.0649
			T_1_	*Y*=27.3199/(1+exp(6.4432-0.335064*t*))	0.9995	25.7874	15.2992	7.8612	2.6270	1.0594	2.2885	19.2298
			T_2_	*Y*=30.4738/(1+exp(6.1855-0.318768*t*))	0.9999	26.2973	15.2729	8.2631	2.7613	1.1588	2.4285	19.4044
			T_3_	*Y*=27.8256/(1+exp(4.8744-0.258264*t*))	0.9994	27.3814	13.7743	10.1989	3.4082	1.0162	1.7966	18.8737

	Sumai 188	WS_7_	CK	*Y*=37.0025/(1+exp(4.4644-0.244162*t*))	0.9951	27.2836	12.8906	10.7879	3.6051	1.3562	2.2587	18.2846
			T_1_	*Y*=38.5689/(1+exp(4.2625-0.233686*t*))	0.9935	27.6428	12.6045	11.2715	3.7667	1.3953	2.2533	18.2403
			T_2_	*Y*=41.0969/(1+exp(4.2318-0.230217*t*))	0.9936	27.9259	12.6611	11.4414	3.8235	1.4716	2.3653	18.3818
			T_3_	*Y*=38.9439/(1+exp(4.2545-0.234300*t*))	0.9952	27.5362	12.5373	11.2420	3.7568	1.4143	2.2811	18.1583
		WS_11_	CK	*Y*=34.9371/(1+exp(4.9973-0.262400*t*))	0.9935	27.4182	14.0255	10.0381	3.3545	1.2742	2.2919	19.0446
			T_1_	*Y*=35.7117/(1+exp(5.2951-0.284353*t*))	0.9902	26.3487	13.9900	9.2631	3.0955	1.3554	2.5387	18.6216
			T_2_	*Y*=36.2078/(1+exp(4.9271-0.264143*t*))	0.9914	26.9715	13.6672	9.9719	3.3324	1.3424	2.3910	18.6532
			T_3_	*Y*=36.3340/(1+exp(4.8815-0.260203*t*))	0.9931	27.2046	13.6989	10.1229	3.3828	1.3356	2.3636	18.7604
		WS_15_	CK	*Y*=25.2967/(1+exp(3.6929-0.199288*t*))	0.9974	29.5558	11.9219	13.2171	4.4168	0.8559	1.2603	18.5305
			T_1_	*Y*=29.2296/(1+exp(3.5198-0.181609*t*))	0.9999	31.4799	12.1294	14.5037	4.8468	0.9285	1.3271	19.3812
			T_2_	*Y*=29.8813/(1+exp(3.8062-0.202551*t*))	0.9995	29.6391	12.2893	13.0041	4.3457	1.0082	1.5131	18.7913
			T_3_	*Y*=29.3759/(1+exp(3.9273-0.205097*t*))	0.9995	29.8616	12.7271	12.8427	4.2917	0.9837	1.5062	19.1485

2022	Yangmai 18	WS_7_	CK	*Y*=46.5062/(1+exp(3.6992-0.191187*t*))	0.9988	30.8411	12.4601	13.7771	4.6040	1.5079	2.2228	19.3486
			T_1_	*Y*=51.0984/(1+exp(3.5655-0.181740*t*))	0.9985	31.7086	12.3721	14.4932	4.8433	1.6115	2.3217	19.6187
			T_2_	*Y*=50.8789/(1+exp(3.6187-0.196395*t*))	0.9987	29.6134	11.7197	13.4117	4.4819	1.7181	2.4981	18.4256
			T_3_	*Y*=51.1861/(1+exp(3.5978-0.189887*t*))	0.9992	30.5183	12.0114	13.8714	4.6355	1.6772	2.4299	18.9471
		WS_11_	CK	*Y*=31.5899/(1+exp(4.6557-0.276685*t*))	0.9973	24.7680	12.0668	9.5199	3.1813	1.2754	2.1851	16.8267
			T_1_	*Y*=35.8646/(1+exp(4.0393-0.231770*t*))	0.9912	26.9082	11.7457	11.3647	3.7978	1.3328	2.0781	17.4281
			T_2_	*Y*=38.8952/(1+exp(3.8009-0.222926*t*))	0.9920	26.9063	11.1423	11.8156	3.9485	1.4456	2.1677	17.0501
			T_3_	*Y*=36.6372/(1+exp(3.9614-0.235234*t*))	0.9911	26.1808	11.2416	11.1974	3.7419	1.3994	2.1546	16.8403
		WS_15_	CK	*Y*=23.9258/(1+exp(3.5457-0.238614*t*))	0.9999	24.0678	9.3402	11.0387	3.6889	0.9941	1.4273	14.8596
			T_1_	*Y*=26.3812/(1+exp(1.8507-0.153667*t*))	0.9985	26.3422	3.4731	17.1410	5.7281	1.0015	1.0135	12.0436
			T_2_	*Y*=35.5361/(1+exp(2.3404-0.155732*t*))	0.9999	29.1374	6.5715	16.9137	5.6522	1.2196	1.3835	15.0284
			T_3_	*Y*=32.4776/(1+exp(1.9379-0.137511*t*))	0.9999	30.0712	4.5153	19.1548	6.4011	1.0800	1.1165	14.0927

	Sumai 188	WS_7_	CK	*Y*=46.9604/(1+exp(3.7339-0.189572*t*))	0.9981	31.2869	12.7492	13.8945	4.6432	1.5010	2.2256	19.6965
			T_1_	*Y*=47.6664/(1+exp(3.5938-0.189730*t*))	0.9983	30.5225	12.0002	13.8829	4.6394	1.5617	2.2609	18.9417
			T_2_	*Y*=48.3420/(1+exp(3.7755-0.210788*t*))	0.9978	28.3352	11.6634	12.4960	4.1759	1.7061	2.5475	17.9114
			T_3_	*Y*=47.4846/(1+exp(3.6954-0.207033*t*))	0.9962	28.4622	11.4880	12.7226	4.2516	1.6683	2.4577	17.8493
		WS_11_	CK	*Y*=30.2138/(1+exp(4.0763-0.237924*t*))	0.9947	26.3678	11.5974	11.0708	3.6996	1.1459	1.7971	17.1328
			T_1_	*Y*=32.5974/(1+exp(3.7223-0.219752*t*))	0.9930	26.9373	10.9455	11.9862	4.0055	1.2101	1.7908	16.9386
			T_2_	*Y*=35.1059/(1+exp(3.5807-0.218164*t*))	0.9954	26.2929	10.3012	11.9862	4.0055	1.3352	1.9286	16.2943
			T_3_	*Y*=34.5883/(1+exp(3.5672-0.211169*t*))	0.9965	27.2977	10.6559	12.4734	4.1683	1.2671	1.8260	16.8926
		WS_15_	CK	*Y*=21.7192/(1+exp(2.9027-0.164092*t*))	0.9994	31.0797	9.6635	16.0520	5.3642	0.6988	0.8910	17.6895
		T_1_	*Y*=26.4782/(1+exp(2.6624-0.134967*t*))	0.9992	36.0060	9.9684	19.5159	6.5218	0.7354	0.8934	19.7263
		T_2_	*Y*=29.4422/(1+exp(2.4965-0.134233*t*))	0.9997	34.9670	8.7870	19.6226	6.5574	0.8420	0.9880	18.5983
		T_3_	*Y*=27.9205/(1+exp(2.3131-0.126460*t*))	0.9999	35.6660	7.8768	20.8287	6.9605	0.7828	0.8827	18.2912

WS_7_, WS_11,_ and WS_15_ respectively represent treatments under waterlogging and shading for 7, 11, and 15 days after anthesis. Shade nets with a shading rate of about 45% were used for shading, and waterlogging preserves about 2 cm of moisture on the surface of the soil. CK refers to the control sprayed with distilled water, T_1_, T_2_, and T_3_ refer to 6-BA sprayed at a concentration of 15, 25, and 35 mg·L^−1^, respectively. Y is the 1000-grain weight of the observed grain. t is the number of days from anthesis until observation. D is the effective days of grouting. D_1_, D_2_, and D_3_ are the duration of grain filling increasing stage, rapid increasing stage, and slow increasing stage respectively. V_mean_ is the average grouting rate, V_max_ is the maximum grouting rate, T_max_ is the occurrence time of the maximum grouting rate. Each value is expressed as mean ± SE (n = 3).

### 3.6 Effect of spraying 6-BA after WS on yield and contributing factors

As shown in **Table 5**, the longer the duration of WS, the lower the number of grains per spike, the thousand-grain weight, and the yield. The different cultivars, treatment durations of WS, and spraying concentrations of 6-BA significantly affected the thousand-grain weight and yield from wheat. Moreover, under the same treatment duration of WS, compared with the control, the application of 6-BA after WS caused a remarkable improvement in the thousand-grain weight and yield in both varieties (*p<* 0.05); however, there were little difference in the spike numbers and kernel numbers per spike. Of the three 6-BA concentrations, spraying 25 mg·L^−1^ of the 6-BA solution had the greatest effect on the thousand-grain weight and yield. Take the results of the wheat growing season from 2020 to 2021 as an example; in Yangmai18, spraying 25 mg·L^−1^ of the 6-BA solution following 7, 11, and 15 days of WS caused an increase in the thousand-grain weight by 5.06%, 7.97%, and 30.52%, and an increase in yield of 9.43%, 18.23%, and 28.15% compared with the control, respectively. Meanwhile, in Sumai 188, the thousand-grain weight increased by 11.21%, 7.23%, and 17.73%, while yield increased by 15.64%, 8.93%, and 24.89%, respectively. Taken together, these findings suggest that the application of 6-BA had the greatest influence on yield after 15 days of WS.

**Table 5:** The effect of exogenous 6-BA on wheat yield and its components under waterlogging and shading.

Cultivar	Treatment		2020–2021				2021–2022			
			Spikes(10^4^ per ha)	Kernel numbersper spike	Thousand grainweight (g)	Grain yield(kg·ha^−1^)	Spikes(10^4^ per ha)	Kernel numbersper spike	Thousand grainweight (g)	Grain yield(kg·ha^−1^)
Yangmai 18	WS_7_	CK	440.22a ± 15.28	39.67a ± 1.20	40.48c ± 0.44	7053.76c ± 55.74	483.33a ± 14.53	42.00a ± 1.53	44.00c ± 0.21	8935.57b ± 467.73
		T_1_	440.22a ± 5.78	41.33a ± 0.88	40.78bc ± 0.33	7415.81b ± 26.06	486.67a ± 6.67	42.00a ± 1.53	48.07b ± 0.07	9828.73ab ± 426.56
		T_2_	450.23a ± 5.78	40.33a ± 0.88	42.53a ± 0.16	7719.05a ± 58.21	506.67a ± 8.82	40.67a ± 0.88	49.57a ± 0.38	10206.91a ± 160.61
		T_3_	443.56a ± 3.34	40.67a ± 0.67	41.72ab ± 0.06	7523.59ab ± 95.60	483.33a ± 14.53	42.33a ± 0.33	49.27a ± 0.24	10074.29ab ± 194.10
	WS_11_	CK	416.88a ± 6.67	39.33a ± 0.88	38.53c ± 0.40	6313.01c ± 26.72	493.33a ± 13.33	38.67a ± 1.20	31.87c ± 0.19	6070.35c ± 108.35
		T_1_	433.55a ± 8.82	39.00a ± 0.58	39.27bc ± 0.34	6634.99b ± 44.12	500.00a ± 10.00	39.33a ± 0.88	36.13b ± 0.26	7102.46b ± 143.81
		T_2_	460.23b ± 5.78	39.00a ± 0.58	41.60a ± 0.10	7463.93a ± 12.66	506.67a ± 8.82	40.33a ± 1.20	38.93a ± 0.24	7954.47a ± 254.27
		T_3_	433.55a ± 8.82	40.33a ± 0.88	40.00b ± 0.18	6989.16ab ± 78.70	500.00a ± 5.77	40.33a ± 1.20	37.03b ± 0.46	7466.85ab ± 236.78
	WS_15_	CK	450.23a ± 10.01	37.33a ± 0.88	25.03c ± 0.88	4198.41d ± 29.21	496.67a ± 18.56	36.00b ± 0.58	23.73d ± 0.15	4238.78c ± 103.67
		T_1_	470.24a ± 5.77	36.33a ± 0.88	28.33b ± 0.92	4832.51b ± 48.82	570.00a ± 15.28	38.00ab ± 0.58	25.57c ± 0.03	5538.66b ± 186.98
		T_2_	466.90a ± 12.02	35.33a ± 0.88	32.67a ± 0.65	5380.31a ± 33.70	533.33a ± 37.12	38.33a ± 0.67	34.00a ± 0.31	6962.96a ± 571.19
		T_3_	453.57a ± 6.67	36.33a ± 0.33	28.42b ± 0.31	4681.22c ± 33.75	526.67a ± 39.30	37.00ab ± 0.58	30.73b ± 0.07	5990.28ab ± 469.63
Sumai 188	WS_7_	CK	423.55a ± 8.82	36.33a ± 0.67	36.75c ± 0.21	5651.24c ± 47.26	496.67a ± 6.67	38.00a ± 0.58	43.93c ± 0.37	8287.86c ± 35.83
		T_1_	440.22a ± 5.78	36.33a ± 0.33	38.45b ± 0.18	6148.49b ± 39.01	496.67a ± 6.67	39.33a ± 0.88	45.50b ± 0.17	8884.36b ± 128.31
		T_2_	440.22a ± 5.78	36.33a ± 0.33	40.87a ± 0.25	6534.98a ± 52.86	503.33a ± 8.82	39.67a ± 0.33	46.80a ± 0.57	9339.07a ± 26.03
		T_3_	440.22a ± 5.78	36.00a ± 0.58	38.38b ± 0.32	6080.64b ± 66.04	490.00a ± 5.77	39.67a ± 0.67	46.10ab ± 0.17	8958.93b ± 149.49
	WS_11_	CK	426.88a ± 3.34	36.00a ± 0.58	35.28c ± 0.10	5421.71c ± 82.75	503.33a ± 3.33	40.33a ± 1.20	30.20c ± 0.15	6132.50c ± 212.73
		T_1_	433.56a ± 8.82	36.67a ± 0.88	36.30b ± 0.10	5764.90ab ± 19.75	496.67a ± 6.67	39.67a ± 0.88	32.63b ± 0.27	6424.44bc ± 24.21
		T_2_	430.22a ± 11.55	36.33a ± 0.88	37.83a ± 0.15	5905.69a ± 37.28	496.67a ± 8.82	41.00a ± 0.58	35.00a ± 0.46	7125.16a ± 144.63
		T_3_	433.55a ± 6.67	36.00a ± 0.58	36.43b ± 0.13	5683.77b ± 35.63	493.33a ± 12.02	39.00a ± 1.00	34.37a ± 0.22	6604.31b ± 66.58
	WS_15_	CK	420.21b ± 5.78	37.00b ± 0.58	24.53d ± 0.25	3812.93c ± 46.25	490.00a ± 5.77	32.67b ± 1.20	20.57d ± 0.35	3291.25c ± 128.13
		T_1_	426.88a ± 3.34	38.67a ± 0.33	27.62c ± 0.21	4558.34b ± 60.82	500.00a ± 5.77	35.33a ± 0.33	23.43c ± 0.12	4140.52b ± 82.31
		T_2_	430.21a ± 5.78	38.33ab ± 0.33	28.88a ± 0.07	4762.15a ± 23.67	496.67a ± 12.02	37.00a ± 0.58	26.47a ± 0.34	4859.88a ± 69.60
		T_3_	433.55a ± 8.82	38.00ab ± 0.58	28.20b ± 0.09	4643.08ab ± 29.39	493.33a ± 6.67	36.00a ± 0.58	24.90b ± 0.06	4423.73b ± 122.92
Significance level			Spikes (10^4^ per ha)	Kernel numbers per spike	Thousand grain weight (g)	Grain yield (kg·ha^−1^)	Spikes (10^4^ per ha)	Kernel numbers per spike	Thousand grain weight (g)	Grain yield (kg·ha^−1^)
Cultivar (C)			**	**	**	**	ns	**	**	**
Treatment duration (D)			*	**	**	**	*	**	**	**
Concentrations of 6-BA (T)			**	ns	**	**	ns	*	**	**
C×D			**	**	**	**	*	**	**	**
C×T			ns	ns	ns	**	ns	ns	**	*
D×T			ns	ns	**	**	ns	ns	**	ns
C×D×T			ns	ns	**	**	ns	ns	**	ns

WS_7_, WS_11_, and WS_15_ respectively represent treatments under waterlogging and shading for 7, 11, and 15 days after anthesis. Shade nets with a shading rate of about 45% were used for shading, and waterlogging preserves about 2 cm of moisture on the surface of the soil. CK refers to the control sprayed with distilled water; T_1_, T_2_, and T_3_ refer to 6-BA sprayed at a concentration of 15, 25, and 35 mg·L^−1^, respectively. Each value is expressed as mean ± SE (n = 3). Different letters labeled after the value among the treatments with different concentrations of 6-BA refer to significant differences at p< 0.05. ** represents significant differences at the 1% level. * represents significant differences at the 5% level. ns represents no significant difference.

## 4 Discussion

### 4.1 Effects of spraying 6-BA after WS on photosynthetic parameters and photosynthetic product transport

Studies have shown that improvements in the photosynthetic area of functional leaves, the photosynthetic duration, and the photosynthetic capacity can improve the grain yield of crops (Parry et al., 2011). Studies have shown that waterlogging accelerates the leaf senescence in wheat, especially under soil compaction, thereby significantly reducing leaf SPAD values as well as *P*_n_, *G*_s_, and *T*_r_, which affects the overall photosynthetic capacity (Wu W. et al., 2018b; Wu X. et al., 2018a). Similarly, insufficient light is also known to limit photosynthesis in wheat (Shimoda and Sugikawa, 2020). This study also found that the *P*_n_ of wheat flag leaves decreased rapidly with the increase of WS treatment duration, especially after 15 days of WS treatment. However, the self-regulation effect of wheat can only resist the damage caused by WS stress in a short time (Liu et al., 2007), thus taking flag leaf photosynthesis at a low level under the 15 days of WS. Meanwhile, studies have also shown that spraying the 6-BA solution before waterlogging can increase the photosynthetic rate of the flag leaves, slowing down the rate of plant senescence, and reducing yield losses (Wang X. et al., 2020a). Notably, maintaining the photosynthetic rate of flag leaves after anthesis was found to effectively increase biomass and yield (Luo et al., 2006; Chen et al., 2010). In this study, spraying the 6-BA solution after WS caused a significant increase in the *P*_n_, *G*_s_, and *T*_r_ of the flag leaves, and a reduction in the *C*_i_. These findings suggest that the stomatal limitations of photosynthesis under WS was relieved by 6-BA, elevating the photosynthesis of wheat flag leaf. In line with this, maintaining a longer photosynthetically active period during grain filling, which allows the transfer of more assimilates into the grains, was previously found to improve resistance to abiotic stress after anthesis (Chen et al., 2010). Therefore, in this study, spraying 6-BA prolonged the photosynthetic activity period of wheat leaves after anthesis, especially under the treatment of WS for 7 days; and spraying 6-BA prolonged the duration of grain filling; the photosynthetic rate of flag leaves of wheat decreased slowly, which increased the amount of photosynthetic products produced by leaves into grains. Moreover, spraying 6-BA can alleviate the effects of waterlogging on the chloroplast and mitochondrial structure, thereby increasing the chlorophyll content and improving the photosynthetic performance of maize after waterlogging (Ren et al., 2017). These results confirmed that spraying 6-BA could improve the photosynthetic capacity of leaves.

After the wheat leaves absorbed ^13^CO_2_, we found that the ability of flag leaves to assimilate CO_2_ improved after spraying the 6-BA solution, and the accumulation of photosynthetic products in grains also increased. 6-BA improved the ability of the flag leaves to assimilate CO_2_, thereby increasing the transportation rate of assimilates. This may be because exogenous 6-BA reduces the damage to the photosynthetic system, improving the assimilation efficiency of CO_2_, which allows the generation of more carbohydrates, thus meeting the needs of plant growth (Ding et al., 2013). Therefore, the results of this study showed that spraying 6-BA could not only improve the photosynthetic capacity of flag leaves but also alleviate the transport process of carbohydrates generated by flag leaves to grains, and this process was also related to the transport of photosynthates in stem and stem sheath. It was found that 7 days after spraying the 6-BA solution, the contents of soluble sugar and starch in different organs of maize (leaves, sheaths, stems and young corn cobs) increased, and 6-BA could significantly improve the translocation rate of photosynthetic products from maize leaves to maize grains (Yang et al., 2019).

### 4.2 Effects of spraying 6-BA after WS on starch granules and starch content

Wheat grain starch exists as starch granules that differ in shape, size, composition, and properties (Shewry, 2010; Vamadevan, 2013; Guo et al., 2019; Yan et al., 2021). Studies have shown that WS reduces the number and volume of A-type starch granules (Li et al., 2020), with deformation of the granules under stress (Wang and Copeland, 2012). However, 6-BA can restore the adverse effects of ethephon on the number and size of starch granules and increase the storage capacity of grains (Liu et al., 2018). In this study, spraying 6-BA after WS could increase the volume of A-type starch granules and reduce the deformation of B-type starch granules, which indicated that spraying 6-BA can alleviate the formation and development of starch granules.

The enrichment process of wheat grains under WS is largely related to the accumulation of starch in the endosperm (Tomlinson and Denyer, 2003). The content and ratio of amylose to amylopectin has an effect on starch structure, gelatinization, and thermal properties (Wang H. et al., 2020b). 6-BA and ABA are the most potent phytohormones in terms of increased biomass and starch accumulation (Liu et al., 2019). In this study, in contrast with the control, the application of 6-BA significantly increased the starch content and starch accumulation rate in grains, and alleviated the effect of WS stress on starch accumulation. The enhanced photosynthetic capacity of wheat after spraying 6-BA increased the photosynthate produced by flag leaves, which improved the amount of carbohydrates transported to grains and resulted in the increase of starch accumulation. Moreover, the application of 6-BA was found to increase the content of starch in various organs, promoting the transport and subsequent accumulation of carbohydrates in the grains (Yang et al., 2019). The activity of starch-related enzymes is able to regulate the starch synthesis rate, thereby affecting carbohydrate synthesis (Ran et al., 2020; Wang et al., 2021); however, spraying the 6-BA solution can improve the activity of enzymes related to starch synthesis, which causes an increase in starch accumulation (Hsu et al., 2014; Luo et al., 2015; Wang et al., 2022).

### 4.3 Effects of spraying 6-BA after WS on grain filling and yield

**Figure 8:**
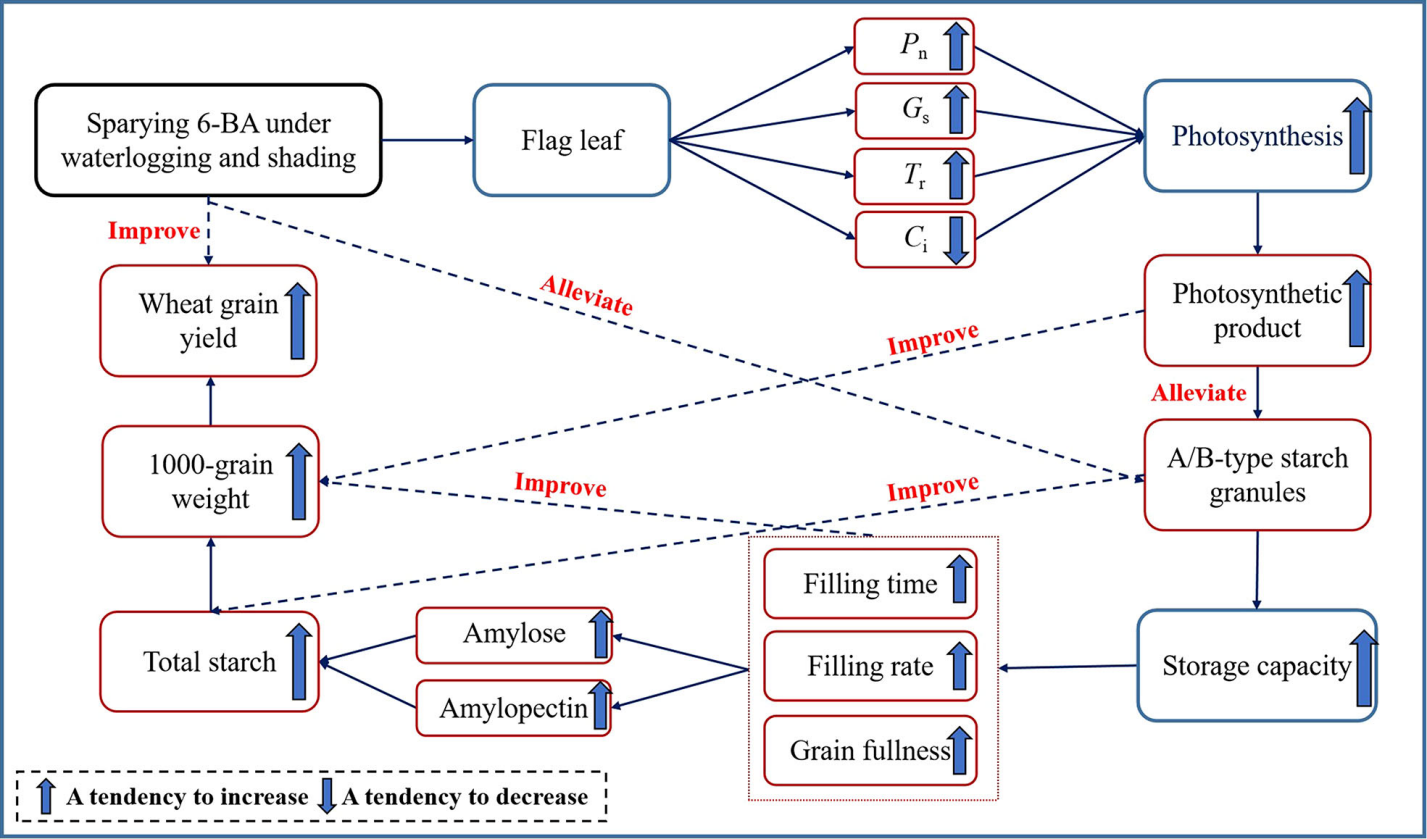
Analysis of the ways of spraying 6-BA to improve wheat yield under waterlogging and shading after anthesis.

The rate and duration of grain filling are the decisive factors of the final grain weight of wheat kernels (Duguid and Br Lé-Babel, 1994; Gelang et al., 2000). Previous studies revealed a significant increase in wheat grain weight and yield with increasing waterlogging (Arata et al., 2019; Ding et al., 2020), while shading treatment caused a significant drop in wheat grain yield (Yang et al., 2020). Meanwhile, studies have also shown that spraying the 6-BA solution before shading can delay the senescence process of flag leaves under shading treatment, improving dry matter accumulation and, ultimately, grain yield (Li et al., 2019). In this study, the application of 6-BA after WS stress could prolong the photosynthetic activity period of wheat leaves after anthesis, which is propitious to the accumulation and transport of photosynthetic products, and thus increase grain weight and grain yield (**Figure 8**). These findings are related to the fact that exogenous 6-BA can delay leaf senescence, increase the chlorophyll content, and improve photosynthetic performance, all of which effectively enhance the grain-filling characteristics and photosynthesis of crops under waterlogging stress, ultimately having a significant effect on yield (Ren et al., 2016).

The grain filling process is directly affected by the storage capacity, and the number of starch granule can affect the grain-filling, and finally affect the dry matter accumulation in the grain, thereby affecting the yield (Tetlow and Emes, 2017; Zheng et al., 2017; Xie et al., 2018). In this study, spraying 6-BA can alleviate the adverse influence of WS on the formation and development of wheat starch granules, to further expand the storage capacity of wheat grains after WS and increase the duration and rate of grain filling and the fullness degree of grains. However, the duration and rate of grain filling were positively correlated with the grain weight (Dias and Lidon, 2009), so 6-BA application eventually improved wheat grain weight and yield (**Figure 8**).

## 5 Conclusions

Exogenous spraying of the 6-BA solution could improve photosynthesis in the flag leaves and alleviate the negative influence on starch granules, increase the content of starch and its components, and improve the duration and rate of grain filling, thus resulting in improvement of grain weight. In addition, under the conditions of this study, spraying 25 mg·L^−1^ of the 6-BA solution had an optimal effect with three different spray concentrations.

## Data availability statement

The original contributions presented in the study are included in the article/supplementary material. Further inquiries can be directed to the corresponding author.

## Author contributions

WZ and ZH designed the experiment. WZ and BW initiated statistical analysis and drafted the manuscript. BW, AZ, and QZ performed the experiments and determined related data. YL and LL contributed to the experiments proceeding and data interpretation. SM and YF assisted in polishing the manuscript. All authors contributed to the article and approved the submitted version.

## Funding

This work was supported by the Key Research and Development Plan of Anhui Province (grant numbers 202204c06020040), the University Natural Science Research Project of Anhui Province (grant numbers YJS20210251), and the National Key Research and Development Plan of China (grant numbers 2017YFD0300205).

## Conflict of interest

The authors declare that the research was conducted in the absence of any commercial or financial relationships that could be construed as a potential conflict of interest.

## Publisher’s note

All claims expressed in this article are solely those of the authors and do not necessarily represent those of their affiliated organizations, or those of the publisher, the editors and the reviewers. Any product that may be evaluated in this article, or claim that may be made by its manufacturer, is not guaranteed or endorsed by the publisher.
